# The Effects of Temperature and Body Mass on Jump Performance of the Locust *Locusta migratoria*

**DOI:** 10.1371/journal.pone.0072471

**Published:** 2013-08-13

**Authors:** Edward P. Snelling, Christie L. Becker, Roger S. Seymour

**Affiliations:** School of Earth and Environmental Sciences, University of Adelaide, Adelaide, South Australia, Australia; The University of Wollongong, Australia

## Abstract

Locusts jump by rapidly releasing energy from cuticular springs built into the hind femur that deform when the femur muscle contracts. This study is the first to examine the effect of temperature on jump energy at each life stage of any orthopteran. Ballistics and high-speed cinematography were used to quantify the energy, distance, and take-off angle of the jump at 15, 25, and 35°C in the locust *Locusta migratoria*. Allometric analysis across the five juvenile stages at 35°C reveals that jump distance (*D*; m) scales with body mass (*M*; g) according to the power equation *D* = 0.35*M* ^0.17±0.08 (95% CI)^, jump take-off angle (*A*; degrees) scales as *A* = 52.5*M* ^0.00±0.06^, and jump energy (*E*; mJ per jump) scales as *E* = 1.91*M* ^1.14±0.09^. Temperature has no significant effect on the exponent of these relationships, and only a modest effect on the elevation, with an overall Q_10_ of 1.08 for jump distance and 1.09 for jump energy. On average, adults jump 87% farther and with 74% more energy than predicted based on juvenile scaling data. The positive allometric scaling of jump distance and jump energy across the juvenile life stages is likely facilitated by the concomitant relative increase in the total length (*L*_f+t_; mm) of the femur and tibia of the hind leg, *L*_f+t_ = 34.9*M* ^0.37±0.02^. The weak temperature-dependence of jump performance can be traced to the maximum tension of the hind femur muscle and the energy storage capacity of the femur's cuticular springs. The disproportionately greater jump energy and jump distance of adults is associated with relatively longer (12%) legs and a relatively larger (11%) femur muscle cross-sectional area, which could allow more strain loading into the femur's cuticular springs. Augmented jump performance in volant adult locusts achieves the take-off velocity required to initiate flight.

## Introduction

Jumping is a form of locomotion used by various groups of vertebrate and invertebrate animals [1]. Jumping has become highly specialised in the locust, and represents the primary means of locomotion for juveniles, while in the adult it provides the thrust necessary to initiate flight [2]. Both juvenile and adult locusts circumvent contractile limitations of muscle by using an energy storage system built into the hind limb, which allows jumps that are of both high force and high velocity [3]. The process of the locust jump begins with the contraction of the small flexor muscle contained within the hind femur, which pulls the tibia almost parallel to the femur. The flexor and large extensor muscles then co-contract, and with the tibia remaining fully flexed owing to a catch mechanism, stress energy is transferred to the femur's internal apodome structures and the semi-lunar processes where it is stored in the form of strain energy. The flexor then suddenly relaxes which frees the tibia and causes the rapid release of energy from the deformed cuticular elements to produce a rapid extension of the tibia [4], [5]. By storing and releasing energy in this manner, the effective power output of the jump is amplified by about 10-times above that generated by the muscle [3].

The energy of the locust jump can be described using ballistics, according to Eq. (1): 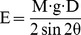
where *E* is the energy (mJ = g m^2^ s^−2^), *M* is body mass (g), *g* is acceleration due to gravity (9.81 m s^−2^), *D* is jump distance (m), and θ is jump take-off angle to the horizontal. It is evident from Eq. (1) that jump energy will be significantly affected by the c.a. 50-fold increase in body mass that occurs throughout locust development, as well as any variation in jump distance or jump take-off angle that could occur through successive life stages. These variations can be investigated using an allometric analysis, which takes the general form of a power equation, *y* = a*M* ^b^, where *y* is the variable being investigated, a is the coefficient (elevation), and b is the scaling exponent (the slope of the log-transformed equation). When the principal of allometric cancellation is applied to this analysis [6], the null hypothesis is that jump energy scales throughout the locust life cycle in direct proportion to body mass (∝*M* ^1^), assuming that *g*, *D* and θ are constants (∝*M* ^0^). A.V. Hill also hypothesised that maximum jump distance should not vary among geometrically similar animals of different body mass [7]. A caveat here is the assumption that locusts maintain geometric similarity throughout the life cycle, since any increase in the hind leg's relative length or femur muscle cross-sectional area could increase jump distance [1], [8]. If leg length or muscle cross-sectional area increases faster than geometric similarity (∝*M* ^0.33^ and *M* ^0.67^, respectively) during locust development, then jump energy and jump distance should show “positive allometry”, with exponents greater than 1 and 0, respectively. Positive allometry in fact appears in locusts of the genus *Schistocerca* [2], [9], [10].

Body temperature could also affect jump performance in the poikilothermic locust. The effect of temperature on performance is often described by an inverted U-shaped curve, whereby performance is optimal at some intermediate temperature range, but declines outside this range [11]. For example, jump distance in the adult house cricket *Acheta domestica* follows an inverted U-shaped relationship with temperatures between 0–45°C, with peak performance occurring around 26–31°C [12]. However, not all jump performance studies involving Orthoptera show the same degree of thermal sensitivity. For example, the adult two-striped grasshopper *Melanoplus bivittatus* has a similar jump distance at 20 and 35°C [13], and jump distance in the adult pygmy grasshopper *Tetrix subulata* increases by only 15% as temperature increases from 15–25°C [14]. The thermal sensitivity of jump energy can be evaluated using the Q_10_ calculation, which is the ratio of change with every 10°C increase in temperature. If jump energy is dependent on the rate at which tension is developed in the muscle, which is generally temperature-sensitive, then a Q_10_ of 2–2.5 would be expected [15]. However, if jump energy is dependent on the absolute maximum tension developed by the muscle, which is often relatively temperature-insensitive, then a Q_10_ of 1.0–1.2 would arise [1], [15]. A low Q_10_ value for jump energy might also be expected because the locust jump is partly a mechanical process involving the rapid release of strain energy from the femur's cuticular springs [1], [16], [17]. While the studies referred to above appear divided over the relative thermal sensitivity of locust jump performance [12]–[14], it is also evident that size effects on temperature sensitivity are unknown. This can be determined from the power equation, by testing for differences in exponent (b) and then elevation (a).

The aim of this study is to use allometry to assess the effects of body mass and temperature on jump distance, jump take-off angle, and jump energy at all six life stages of the locust *Locusta migratoria* to test the hypotheses presented above. Although scaling of jump performance across ontogeny in *Schistocerca*, and temperature dependence of jump distance in adult *A*. *domestica*, *M*. *bivittatus*, and *T*. *subulata* have been determined independently, this is the first study to combine scaling and temperature dependence in all life stages of an orthopteran.

## Materials and Methods

Gregarious-phase locusts *Locusta migratoria* (Linnaeus 1758) came from a breeding colony (35±2°C) at the University of Adelaide [18]. The founding stock was sourced from wild populations in inland eastern Australia, where annual daytime temperatures typically range 10–40°C (http://www.bom.gov.au/climate/data-services/). Jump performance measurements and metathoracic (hind) leg lengths were taken from locusts at all six stages of the life cycle, including adults. Recently moulted locusts (<1-day-old) were not used because their soft cuticle, and potentially relatively small muscle mass, can compromise jump performance [19]. For volant adult locusts, it was necessary to bind the wings with adhesive tape (1–2% body mass) to prevent flight.

Each locust was placed in a controlled temperature room where it was given a minimum of 30 min to equilibrate to one of three experimental temperatures: 15, 25 or 35°C (verified with a mercury thermometer). Each individual was then encouraged to perform 3–5 “escape-type” jumps on a cotton sheet by startling the insect from behind, and when necessary, lightly prodding the tip of the abdomen. The horizontal, straight-line distance between the starting and landing points of each jump (taken midway along the body) was immediately measured to the nearest 0.5 cm using either a 30 or 100 cm ruler, as appropriate. In addition, the initial jump take-off was filmed at 240 frames s^−1^ using a high speed digital video camera (Xacti VPC-FH1, Sanyo Electric Co., Osaka, Japan), which was positioned lateral to the locust, and perpendicular to the predicted direction of the jump. Jumps that deviated more than ±15 degrees from perpendicular were excluded, in order to limit the difference in actual versus perceived take-off angle [20]. Take-off angle was measured to the nearest 0.1 degree by overlaying individual video frames from the start of the jump and using an angle tool in a computer graphics program (CorelDRAW 11, Corel Corp., Ottawa, ON, Canada). Locusts were weighed to 0.1 mg on an analytical balance (AE163, Mettler, Greifensee, Switzerland), and then body mass, jump distance and jump take-off angle were used to calculate energy output for each jump according to Eq. (1), consistent with the methods of earlier studies [9], [10], [16]. The average energy of the 3–5 jumps performed by each locust was then calculated and used in all analyses.

At each experimental temperature, six locusts were used from each of the five juvenile life stages (*N* = 30 juveniles), plus six adult male and six adult female locusts (*N* = 12 adults). No locusts were re-used at different life stages or at different temperatures. Thus, in total, 126 locusts were used for jump performance measurements, consisting of 90 juveniles and 36 adults. The length of the metathoracic femur and tibia (hind leg) was measured to 0.1 mm with digital callipers in every locust used for jump performance measurements (*N* = 126), and in an additional 24 juvenile and 8 adult locusts (*N* = 32). Thus, in total, leg length measurements were taken from 158 locusts, consisting of 114 juveniles and 44 adults.

Mean values are presented with 95% confidence intervals (CI). All other data are expressed allometrically, by taking the log_10_ of the variable and the log_10_ of body mass, and then plotting ordinary least-squares linear regressions. The slopes and intercepts of the regressions were compared with ANCOVA, with body mass as the covariate, according to Zar [21], using GraphPad Prism 5 statistical software (GraphPad Software, La Jolla, CA, USA). When converted to the allometric power equation, the exponent (b) describes the change in the variable as body mass increases throughout ontogeny. The exponents are presented with 95% CI, and if they are statistically indistinguishable, the elevation (a) of the equation is used to describe the effect of temperature on the variable, from which Q_10_ was calculated as the quotient of the elevation values at different temperatures.

## Results

**Figure 1:**
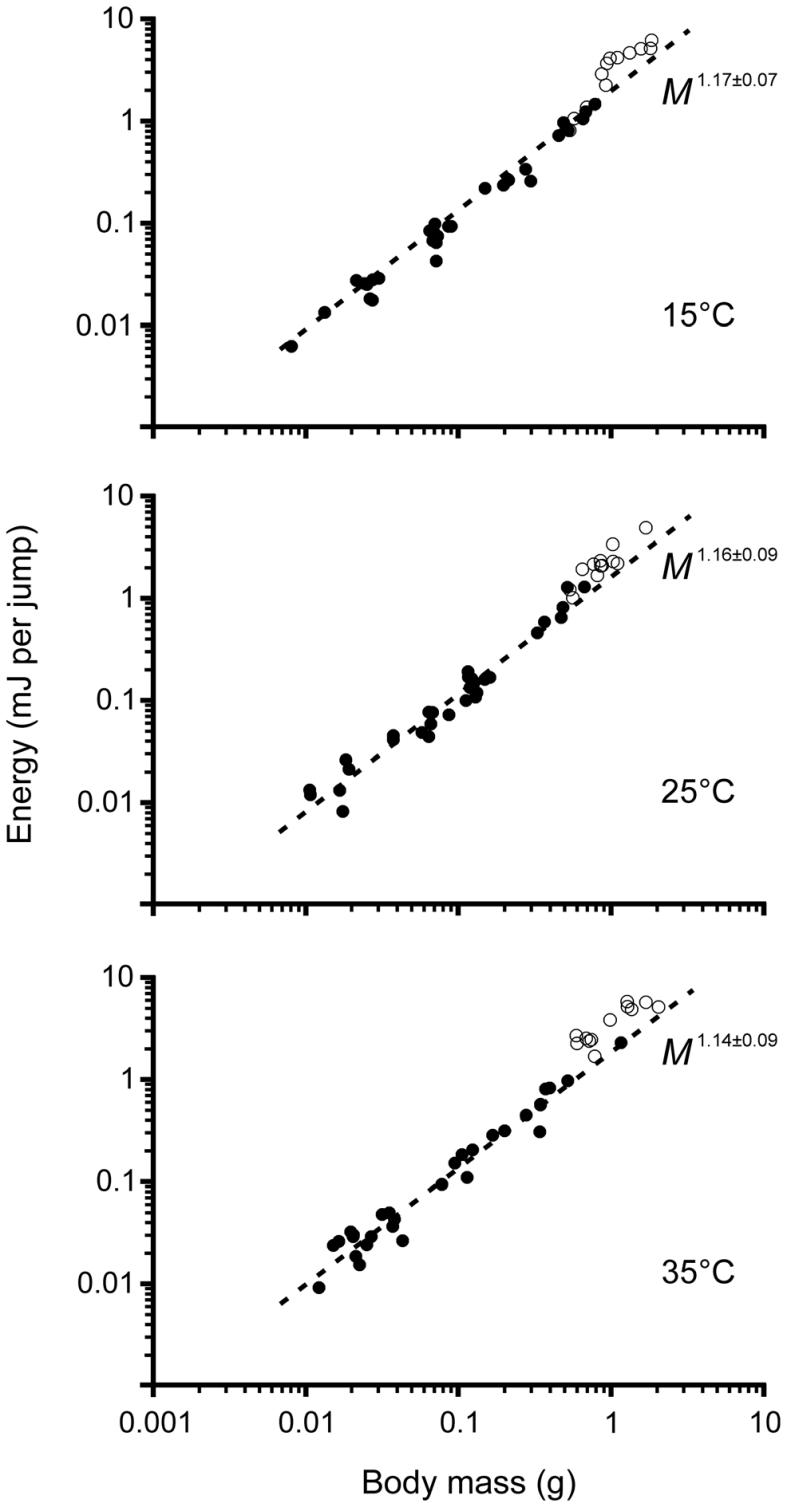
Effect of temperature on the allometric relationship between jump energy and body mass across the five juvenile life stages of *Locusta migratoria* (filled circles; *N* = 30 juvenile individuals per temperature, *N* = 90 juvenile individuals in total). Adult jump energy is also presented (unfilled circles; *N* = 12 adult individuals per temperature, *N* = 36 adult individuals in total), but is not included in the regression due to their disproportionately high values.

Mean values of body mass, jump distance, jump take-off angle, and jump energy at each experimental temperature are provided in Table 1. The scaling of jump energy across five juvenile instars fit allometric equations well, but the data from adults cluster above the line (Fig. 1). The exponents of juvenile jump energy equations do not differ significantly between the three temperatures (ANCOVA, 15 and 25°C, F_1,56_ = 0.07, P = 0.79; 15 and 35°C, F_1,56_ = 0.37, P = 0.55; 25 and 35°C, F_1,56_ = 0.08, P = 0.78; *N* = 30 at each temperature), and so it is possible to calculate a single exponent for all juvenile jump energy data (*M* ^1.15±0.05^). The positive allometric scaling of jump energy in juvenile locusts reflects the finding that juvenile jump distance increases as body mass increases, with a combined exponent across all three temperatures of *M* ^0.16±0.05^, while jump take-off angle in juveniles does not vary with body mass, scaling with a combined exponent of *M* ^0.01±0.02^ (Table 2).

**Table 1:** Effects of life stage and temperature on jump performance of *Locusta migratoria*.

	Life stage	Body mass (g)	Jump distance (m)	Take-off angle (degrees)	Jump energy (mJ per jump)
15°C	First	0.021±0.006	0.17±0.03	45±6	0.019±0.007
	Second	0.051±0.019	0.16±0.05	44±9	0.047±0.024
	Third	0.078±0.008	0.20±0.02	47±5	0.081±0.009
	Fourth	0.23±0.04	0.22±0.03	45±7	0.26±0.03
	Fifth	0.61±0.11	0.31±0.04	47±9	1.03±0.21
	Adult	1.12±0.26	0.56±0.10	41±3	3.40±0.99
25°C	First	0.016±0.003	0.18±0.04	49±9	0.016±0.005
	Second	0.061±0.016	0.17±0.02	55±8	0.057±0.012
	Third	0.103±0.026	0.19±0.03	49±6	0.104±0.030
	Fourth	0.13±0.01	0.22±0.02	52±5	0.16±0.02
	Fifth	0.48±0.10	0.30±0.05	55±9	0.86±0.29
	Adult	0.90±0.17	0.48±0.05	42±3	2.31±0.58
35°C	First	0.018±0.003	0.20±0.06	55±10	0.023±0.007
	Second	0.030±0.006	0.20±0.02	55±8	0.035±0.009
	Third	0.067±0.031	0.19±0.05	56±10	0.082±0.050
	Fourth	0.20±0.07	0.27±0.04	48±9	0.29±0.08
	Fifth	0.52±0.25	0.34±0.05	57±4	1.02±0.52
	Adult	1.06±0.26	0.70±0.08	49±3	3.75±0.88

Data are means ±95% CI calculated from six individuals for each of the five juvenile life stages, and from 12 individuals for the adult life stage. *N* = 30 juvenile and 12 adult individuals per temperature. *N* = 126 individuals in total.

**Table 2:** Effect of temperature on allometric power equations for jump performance of juvenile *Locusta migratoria*.

	Jump distance (m)^NS^,†,‡	Take-off angle (degrees)^NS^,*,†	Jump energy (mJ per jump)^NS^,†,‡
15°C	0.30*M* ^0.17±0.07^ (r^2^ = 0.45)	47.1*M* ^0.02±0.06^ (r^2^ = 0.02)	1.62*M* ^1.17±0.07^ (r^2^ = 0.98)
25°C	0.28*M* ^0.14±0.09^ (r^2^ = 0.28)	53.8*M* ^0.02±0.06^ (r^2^ = 0.02)	1.66*M* ^1.16±0.09^ (r^2^ = 0.96)
35°C	0.35*M* ^0.17±0.08^ (r^2^ = 0.39)	52.5*M* ^0.00±0.06^ (r^2^ = 0.00)	1.91*M* ^1.14±0.09^ (r^2^ = 0.96)

Regressions generated from mean data calculated from six individuals belonging to each of the five juvenile life stages, at each temperature. *N* = 30 juvenile individuals per temperature. *N* = 90 juvenile individuals in total. Equations are in the form *y* = a*M* ^b (±95% CI)^, where *y* is the variable of interest, a is the coefficient (elevation), b is the exponent (slope), and *M* is body mass in grams.

NS Indicates no significant difference in slope between temperatures (ANCOVA, P>0.05);

*indicates a significant difference in elevation between 15 and 25°C (P<0.05);

† indicates a significant difference in elevation between 15 and 35°C (P<0.05);

‡ indicates a significant difference in elevation between 25 and 35°C (P<0.05).

An analysis of the elevations of the juvenile jump energy equations (excluding adults) shows that the intercepts for the 15 and 25°C temperature treatments are statistically indistinguishable (ANCOVA, F_1,57_ = 0.83, P = 0.37), whereas the 35°C temperature has a higher elevation than both the 15 and 25°C treatments (35 and 15°C, F_1,57_ = 12.98, P<0.001; 35 and 25°C, F_1,57_ = 5.96, P = 0.02). Nonetheless, the difference in elevation between temperatures is relatively modest, such that Q_10_ = 1.02 between 15 and 25°C, Q_10_ = 1.15 between 25 and 35°C, and Q_10_ = 1.09 between 15 and 35°C. The modest effect of temperature on jump energy in juvenile locusts reflects the finding that juvenile jump distance is also weakly dependent on temperature, with an overall Q_10_ of 1.08 (Table 2).

Consistent with juvenile locusts, the jump energy of adult locusts at 15°C is statistically indistinguishable from that observed at 25°C (ANOVA with Tukey's *post hoc* test, P>0.05; *N* = 12 adults at each temperature), but at 35°C adult jump energy is significantly higher than at 15 and 25°C (P<0.05). Nonetheless, the difference in adult jump energy between temperatures is once again relatively modest, with an overall Q_10_ of 1.12, which reflects the small effect of temperature on jump distance, where the overall Q_10_ is 1.08. However, at all temperatures adult jump energy is significantly greater relative to body mass compared to the juveniles, and for this reason, adults are treated separately. At 15°C, adult jump energy is 76% greater than predicted from juveniles of the same body mass, and at 25 and 35°C adult jump energy is 55 and 90% greater, respectively (Fig. 1). This reflects the finding that adults jump farther relative to body mass compared to juvenile locusts. At 15°C, adult jump distance is 85% farther than predicted, and at 25 and 35°C adult jump distance is 75 and 102% farther, respectively. The relative difference in jump distance and jump energy are not exactly equivalent to one another, at each respective temperature, because the overall mean jump take-off angle of adults is 44.4±2.0 degrees, whereas in juveniles it is significantly higher, 50.6±2.0 (t-test, t_124_ = 3.5, P<0.001), and this is factored into the calculation for jump energy, Eq. (1).

Metathoracic femur length (*L*_f_; mm) increases with body mass across the juvenile life stages (excluding adults) according to the power equation, *L*_f_ = 18.1*M* ^0.37±0.02^ (r^2^ = 0.91; *N* = 114 juveniles), while tibia length (*L*_t_) increases according to *L*_t_ = 16.7*M* ^0.37±0.02^ (r^2^ = 0.90), such that the combined length of the femur and tibia (*L*_f+t_) scales as *L*_f+t_ = 34.9*M* ^0.37±0.02^ (r^2^ = 0.91). Based on these equations for juveniles, the length of the adult femur, tibia, and combined femur and tibia are all 12% longer than predicted (*N* = 44 adults).

## Discussion

An important finding of this study is that jump energy (∝*M* ^1.15^) in juvenile locusts increases disproportionately with body mass (Fig. 1). If energy were proportional to mass, then all instars would be expected to jump the same distance. However, jump distance scales positively with body mass (∝*M* ^0.16^) with virtually the same exponent above 0.0 as jump energy is above 1.0 (Table 2). Thus a 10 mg first instar jumps 16 cm and a 1 g fifth instar jumps 35 cm at 35°C. This finding is consistent with studies on *Schistocerca* locusts, where jump energy in juveniles scales with an exponent of *M* ^1.11^ [2], and maximum jump distance across juvenile and adult life stages scales as *M* ^0.20–0.22^ [9], [10]. The positive allometric scaling of jump distance and energy observed in the present study could arise partly because smaller instars have a higher frontal area-to-body mass ratio than larger instars, thus making them more susceptible to the effects of aerodynamic drag [22]. However, to some extent, this effect is likely offset by the higher body density of smaller instars compared to larger instars [22], owing to the disproportionate increase in tracheal system volume (∝*M* ^1.30^) that occurs throughout locust development [23]. Certainly the fact that Katz and Gosline [2] arrived at a similar exponent for jump energy (*M* ^1.11^), even though they circumvented the effects of drag using a force plate to calculate kinetic energy, suggests that other factors must contribute strongly to the positive allometry of jump distance and energy. Importantly, our study shows that the length of the hind leg increases disproportionately with body mass (∝*M* ^0.37^) throughout juvenile ontogeny. This is relevant because jump distance is proportional to the distance through which the force acts, which is related directly to limb length [1], [8], [10]. Thus, the relatively longer legs of older juveniles likely provide the means to propel these animals farther and with greater jump energy. Conversely, femur muscle cross-sectional area appears to maintain near-geometric proportionality throughout juvenile development, scaling as *M* ^0.68^, which we derived from the allometric cancellation of juvenile femur muscle volume (∝*M* ^1.05^) [24] and juvenile femur length (∝*M* ^0.37^). The proportional scaling of cross-sectional area implies that the femur muscle's capacity to deform the cuticular elements is unlikely to vary across juvenile ontogeny. Thus, unlike hind limb length, femur muscle cross-sectional area is less likely to contribute to the positive allometry of juvenile jump distance and energy.

Another significant finding of this study is that jump energy and jump distance are only weakly dependent on temperature, such that jump energy has an overall Q_10_ of 1.09 and jump distance has an overall Q_10_ of 1.08 (Fig. 1; Table 2). This is consistent with the finding that temperature has no significant effect on average or maximum jump distance in the adult two-striped grasshopper *Melanoplus bivittatus* [13], and only a modest effect (Q_10_ of 1.15) on jump distance in the adult pygmy grasshopper *Tetrix subulata* [14]. More broadly, these findings are consistent with reports in other insect groups, particularly ants and cockroaches, that minimum cost of transport (mJ g^−1^ m^−1^) is unaffected by temperature [25]–[27]. The modest Q_10_ values for locust jump distance and energy likely arise if temperature has a limited effect on both the maximum tension developed by the femur muscle during contraction [15], [28], and the energy storage capacity of femur's cuticular springs [1], [13], [16]. However, one should be cautious before extrapolating the current findings to temperatures outside the 15–35°C range tested. At higher temperatures, the capacity of the femur muscle to produce tension might decline due to insufficient Ca^2+^ release or insufficient time to generate tension before deactivation processes are initiated, as has been hypothesised for muscle in general [29]. At lower temperatures, the force-generating capacity of individual cross-bridges may start to decline [28], or the time taken to reach maximum tension might become so protracted that the locust initiates the jump early. These factors might explain why the adult house cricket *Acheta domestica* has a relatively comparable jump distance between 20–35°C, but exhibits very poor jump performance between 0–10°C and at 45°C [12].

The third important finding of this study is that jump energy and jump distance of adults are significantly greater than expected of juveniles of the same body mass (Fig. 1). Averaged over all temperatures, adults jump 87% farther and with 74% more energy than predicted from juvenile scaling. The slight mismatch between the relative difference in jump distance and jump energy occurs because the take-off angle in adults (44.4 degrees) is closer than it is in juveniles (50.6 degrees) to the 45 degree optimum angle, which is the trajectory that maximises distance for a given amount of energy according to Eq. (1), or 43 degrees if aerodynamic drag is considered [19]. The reason the overall mean take-off angle of juvenile locusts is somewhat higher than optimal is unclear, although the protocol used to startle the insect from behind could elicit a less efficient escape jump (ratio of energy-to-distance). In any case, the greater jump distance and energy recorded from adult locusts in this study is consistent with adult *Schistocerca americana* locusts where maximum jump distance is approximately 80% farther than predicted from juvenile scaling [9]. The higher jump energy and longer jump distance of adult locusts could be facilitated by the slightly longer (12%) relative length of the adult hind leg, which, as already discussed for juveniles, would allow for increased jump energy by lengthening the distance over which the force acts. In addition, recently published data shows that adult femur muscle volume is 24% larger than that of similarly sized juveniles [24], [30], and if this is combined with the knowledge that adult femur length is 12% longer, the calculated mean cross-sectional area of the femur muscle is 11% larger. As discussed earlier, this is important because a relatively larger muscle cross-section would allow more strain to be loaded into the femur's cuticular springs, thus increasing jump energy. The longer lifespan of the adult life-stage might also allow more time for the cuticular springs to stiffen and augment energy potential [31], [32] and it should also allow the cuticular springs to operate at their functional capacity for longer than is possible in juvenile life-stages, where a decrease in jumping ability occurs around each moult [19]. Functionally, the greater jump energy of adults compared to juveniles relates to the difference in the way juvenile and adult locusts utilise the jump − flightless juvenile life stages jump as the primary mode of locomotion, whereas adult locusts jump to achieve a minimum take-off velocity of 2.5 m s^−1^ required to initiate flight [2], [33]. Kinetic energy (*E*) is related to mass (*M*) and velocity (*v*) according to 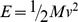. With a mean adult mass of 1.06 g and jump energy of 3.75 mJ at 35°C (Table 1), then initial velocity is 2.66 m s^−1^, which is above the minimum take-off requirement. Fifth instars, however, fall short, with a velocity of 1.98 m s^−1^.

In summary, our investigation into the effects of body mass and temperature on ballistic jump energy at each stage of the locust lifecycle – from a 20 mg first instar to a 1 g adult – reveals three important findings: firstly, that jump energy does not scale in direct proportion to body mass across the five juvenile life stages, but instead exhibits positive allometry, scaling with an overall exponent of *M* ^1.15^; secondly, that jump energy is only weakly dependent on temperature (Q_10_ = 1.09) over the 15–35°C range examined; and thirdly, that the energy of the adult jump is disproportionately greater (74% more energy), relative to body mass, than the juvenile jump, which provides the adult with the initial take-off thrust required for flight initiation.
